# Between Academic Resilience and Burnout: The Moderating Role of Satisfaction on School Context Relationships

**DOI:** 10.3390/ejihpe11030055

**Published:** 2021-07-19

**Authors:** Luciano Romano, Piermarco Consiglio, Giacomo Angelini, Caterina Fiorilli

**Affiliations:** Department of Human Sciences, University of Rome LUMSA, 00193 Rome, Italy; piermarco.consiglio@gmail.com (P.C.); g.angelini@lumsa.it (G.A.); fiorilli@lumsa.it (C.F.)

**Keywords:** academic resilience, school burnout, classmates, teachers, satisfaction, relationships

## Abstract

School burnout is considered an extreme form of maladjustment that can seriously undermine the academic path of students who are affected. Previous studies have focused on possible protective factors, highlighting the role of academic resilience, i.e., the ability to overcome chronic adversity in the school setting. Notwithstanding this, it is equally important to explore the role of the classroom environment and the satisfaction felt by the student toward relationships with teachers and classmates. Therefore, the purpose of the present study was to examine the relationship between academic resilience and burnout and to explore the moderating role of relationship satisfaction with teachers and classmates. A sample of 576 Italian students (Female = 53.1%), aged 14–18 (M = 15.73, SD = 1.56) were involved in the study. Correlations and moderated regressions analyses were conducted to test the hypotheses. The results show academic resilience and satisfaction as inversely related to school burnout. Furthermore, the satisfaction on the relationships with classmates moderated the relation between academic resilience and burnout. Findings were discussed by highlighting the importance of promoting both individual and contextual factors to prevent burnout risk.

## 1. Introduction

School burnout is a syndrome related to overwhelming stress caused by a student’s chronic overexposure to excessively pressing demands that he/she is no longer able to meet [1]. Previous studies have shown that academic resilience can be an influential protective factor toward it [2]. Academically resilient students demonstrate an extreme ability to pick themselves up following particularly traumatic events in their schooling and manage to complete their studies with excellent results in the face of chronic failure [2]. In addition, they are very often able to benefit significantly from the school context in which they are placed, both in terms of the support and relationships they can establish [3,4]. Indeed, as previous authors have pointed out, resilient characteristics come from the interaction among different personal variables with contextual factors. Through their resilience, individuals may face and solve adversities, risks, or traumas. As an essential consequence, resilience involves individual adaptation to the environment and personal growth [5].

Consequently, if the social relationships established in the school context are not highly satisfying, this may undermine the effectiveness of resilience itself towards extreme forms of maladjustment. Conversely, whether one is placed in a highly gratifying context, it is possible to hypothesize that this further enhances the protective action of resilience towards burnout. Therefore, the present study aimed to analyze the association between academic resilience and burnout and explore the moderating role of satisfaction on relationships with teachers and classmates in this relationship.

### 1.1. Academic Resilience as a Personal Resource against School Burnout

High levels of chronic stress in academic contexts can result in school burnout, characterized by emotional exhaustion, a cynical attitude toward school, and feelings of inadequacy as a student [1,6,7]. Several scholars considered emotional exhaustion as the first and core sign of burnout (e.g., [8]). Cynicism, instead, acts as a dysfunctional coping strategy to counteract emotional consumption [9]. Finally, a sense of inadequacy as a student results in a reduced sense of satisfaction and accomplishment toward the school context and the student’s role [1,10]. Previous studies have highlighted that burned-out students are more likely to report poor performance, low personal satisfaction, and disengaged behaviors.

Furthermore, they show low well-being associated with several mental health problems, both during and after the school experience [1,11,12,13,14,15,16,17,18]. Further studies posited that school burnout could occur when taxing and overwhelming external demands exceed the available resources (e.g., [14,19]). Nonetheless, students who benefit from an adequate amount of internal and external resources, such as adequate coping strategies [20], being in a high-achieving peer group [21], and the motivation to pursuit achievement-related goals [22], are more likely to be shielded against the burnout-related consumption process (e.g., [23,24]).

In the same vein, recent studies have focused on the role played by resilient characteristics, deepening how they can effectively protect students from severe forms of maladjustment, such as school burnout [25,26]. Resilience is widely considered a personal capacity to efficiently handle setbacks, challenges, and pressures [27] and adapt despite adverse circumstances [28]. In the academic context, it is defined as the capability to maintain high levels of achievement, motivation, and performance in the face of adverse academic conditions [29]. Resilient students have successful beliefs about themselves and efficient skills: they know how to regulate their actions to achieve their goals, and they see mistakes as a way to improve their skills and knowledge [30,31,32]. Previous studies have shown that academic resilience effectively shields students from experiencing negative emotions derived from excessive academic strain and pressures [33,34,35]. In addition, further research has demonstrated that academically resilient students are more likely to recover from acute and chronic school-related difficulties (i.e., school burnout). Indeed, they are more able to cope with overwhelming school tasks showing higher passion and determination than their low-resilient counterparts [2,29,36,37,38].

### 1.2. The Moderating Role of Students’ Satisfaction on the Relationship with Classmates and Teachers

Students in high school have to deal with new and complex challenges, spending most of their time in a classroom with the same teachers and classmates. Considering the duration of high school in the Italian school system, where the current study was conducted, students have to develop and maintain these relationships for five years, sharing pressures, success, and failures. In addition, during adolescence and as part of their growth process, students need to count on significant others outside the family context to reinforce their beliefs and build their identity [39]. Therefore, high school students’ well-being and development are strictly related to the inner relationships settled with teachers and peers (e.g., [40]).

Further studies have shown that the school context, especially in terms of the classroom climate, plays a pivotal role in contrasting school maladjustment. For instance, several authors have posited that students who rate classroom relationships (e.g., their teachers and peers) as positive and fulfilling might experience a sense of involvement. Consequently, a good classroom climate improves their academic achievements, engagement levels, and chances to carry on their studies [41,42,43]. By contrast, students who experience low or a lack of satisfactory relations with teachers and peers during their academic path are more likely to develop psychological and behavioral problems, both inside and outside of the school context [44,45]. Despite this, what is less studied and worth investigating is how satisfaction on these relationships (i.e., classmates and teachers) interacts with a personal resource such as academic resilience in protecting students from severe forms of maladjustment, such as school burnout.

Indeed, in contrast to the early conceptualization of resilience as a unique trait proper to only a few individuals, the recent literature considers resilience as a learnable process that depends on the interplay among personal characteristics, the environment, and the evaluation of the stressful event [2,46]. Thus, students’ ability to overcome chronic difficulties cannot fail to be related to the school context in which they are included and their satisfaction on classroom relations.

Previous research found that resilient students are more likely to perceive their learning environment as supportive and satisfying than their low-resilient counterparts (e.g., [47]). Furthermore, Dyrbye et al. [48] found that resilient students are less likely to experience burnout when they rate the environment highly satisfying. Further studies have highlighted that resilience in students was associated with higher satisfaction on interactions with teachers and lower burnout [49,50,51].

According to Masten et al. [52], resilient students who rate the quality of their relations with teachers as positive are more able to overcome severe forms of maladjustment. On the other hand, a negative evaluation of the school climate can jeopardize the protective role of students’ resilience, resulting in the onset of stress symptoms and lower levels of well-being [53].

Furthermore, recent research showed that resilient students who report higher satisfaction levels on the support received by their peers are less prone to school maladjustment than their lower satisfied counterparts [54]. Therefore, despite the protective role of resilience, the academic literature suggests that resilient students are less able to cope with school maladjustment whether they do not perceive the relationships with teachers and classmates as supportive and satisfying [52,55,56].

In light of this evidence, it is interesting to understand the protective role of academic resilience towards school burnout and examine the potential moderating role of satisfaction on the relationships established in the classroom (e.g., teachers and classmates).

### 1.3. Aims and Hypotheses

The current study aimed to examine the relationship between academic resilience and school burnout in a sample of Italian high school students by considering the possible moderating role of their satisfaction on the relationship with classmates and teachers. Thus, it was hypothesized that:

**Hypothesis** **1** **(H1).** *Academic resilience, satisfaction on the relationship with classmates, and satisfaction on the relationship with teachers are inversely associated with school burnout;*

**Hypothesis** **2** **(H2).** *Satisfaction on the relationship with teachers would moderate the link between academic resilience and school burnout. We expected that the inverse link between academic resilience and school burnout would be stronger at higher (vs. lower) satisfaction on relationship with teachers.*

**Hypothesis** **3** **(H3).** *Satisfaction on the relationship with classmates would moderate the link between academic resilience and school burnout. In detail, it was expected that high levels (vs. low) of the moderator would boost the protective effect of academic resilience on school burnout.*

## 2. Materials and Methods

### 2.1. Participants

Participants were 576 Italian students (Female = 53.1%), aged 14–18 (M = 15.73, SD = 1.56) and belonged to several high schools located in Northern (29.7%), Central (35.6%), and Southern Italy (34.7%). There were no missing data. The initial sample was composed of 578 students, but due to outliers, two participants were excluded. Students mainly attended a high school focusing on social science and humanities (29.7%), a high school focusing on classical studies (e.g., Greek and Latin) (28.6%), a high school focusing on scientific subjects (16.7%), and a foreign language high school (10.6%).

### 2.2. Instruments

Academic resilience. The academic resilience subscale of the Italian Questionnaire for Anxiety and Resilience (QAR; [57]) was used. It is composed of seven items on a 5-point Likert scale (1 = “Not at all”, 5 = “Totally”). An example of an item is: “I can cope with tension and agitation and get back from the challenging study moments.” In the present study, Cronbach’s alpha was 0.65.

Satisfaction on the relationship with classmates and teachers. To measure students’ satisfaction on the relationship with classmates and teachers, two ad hoc questions on a 4-point Likert scale (1 = “Unsatisfactory”, 4 = “Very satisfactory”) were formulated. The questions were: “How do you evaluate the relationship with your classmates?” and “How do you evaluate the relationship with your teachers?”. The use of single-item scales is a common practice that has been already used in previous studies (e.g., [58,59]).

School burnout. The Italian validated version of the School Burnout Inventory (SBI; [1]) was used to evaluate students’ burnout levels. The scale is composed of nine items on a 6-point Likert scale (1 = “I totally disagree,” 6 = “I totally agree”). Several studies have shown that the scale could be used to measure both the three core dimensions of school burnout separately (i.e., emotional exhaustion, cynicism, and sense of inadequacy as a student) and as a global score [7,15]. The present paper was chosen to measure it as a global score, and Cronbach’s alpha was 0.82.

### 2.3. Procedure

The study was conducted in Italy in February 2020 and adopted a cross-sectional design with a convenience sample. The school council approved the research protocol before starting administrations, and students’ participation was voluntary. Only the students who filled up the informed consent took part in the study. For underage students, only the ones whose parents provided a signed informed consent could be enrolled. Anonymity and confidentiality standards were assured. The participants completed a self-report questionnaire during regular school hours, and it took about 40 min to be completed. A team member was present in case of need and gave all necessary information to fill the questionnaire. The study complied with the Declaration of Helsinki of 1964 and its latest version. The Ethics Committee approval of Lumsa University of Rome, Italy, was obtained before the conduction of the study.

### 2.4. Analysis Plan

In the current study, SPSS v. 21.0 (Statistical Product and Service Solutions, Chicago, IL, USA) was used to perform statistical analyses. First, descriptive statistics, such as skewness and kurtosis, were performed to test the study variables’ adequate normality. Since skewness and kurtosis were not >2, the normal distribution of the study variables was assumed. Furthermore, to verify the associations among the study variables, Pearson correlations were conducted.

Multiple hierarchical regression was conducted to test the link between academic resilience and school burnout and the moderating role of students’ satisfaction on the relationship with classmates and teachers in this association. School burnout was the outcome variable. In Step 1, gender and age were inserted as control variables. In Step 2, academic resilience, satisfaction on the relationship with classmates, and satisfaction on the relationship with teachers were included as predictors. In Step 3, the product between academic resilience and satisfaction on the relationship with classmates and the product between academic resilience and satisfaction on the relationship with teachers were included (interaction terms). Before calculating the interaction term, the single scores were centered on their means, following [60]. Moreover, the results were plotted, and the simple slope analysis was performed using Interaction v. 1.7. [61] to understand the relationship and significance of the predictors on the outcome at different levels (+1/−1 Standard deviation) of the moderator.

## 3. Results

### 3.1. Descriptive Statistics and Correlations

Table 1 shows the means, standard deviations, skewness, kurtosis, and correlation matrix of the studied variables. Gender was coded as a dummy variable, with Male = 0 and Female = 1.

Results from the correlation matrix highlighted that while males were more satisfied on their relationship with classmates, female students were more satisfied on their relationship with teachers. Furthermore, younger students were also more resilient and satisfied on their relationships with classmates and teachers, and older students were more inclined to burnout than their younger peers. Moreover, academic resilience (*r* = −0.48, *p* < 0.01), satisfaction on relationships with classmates (*r* = −0.18, *p* < 0.01), and teachers (*r* = −0.45, *p* < 0.01) were negatively associated with school burnout.

### 3.2. Moderation Analysis Results

Table 2 reports the results from the multiple hierarchical regression analyses with the interaction terms.

**Figure 1:**
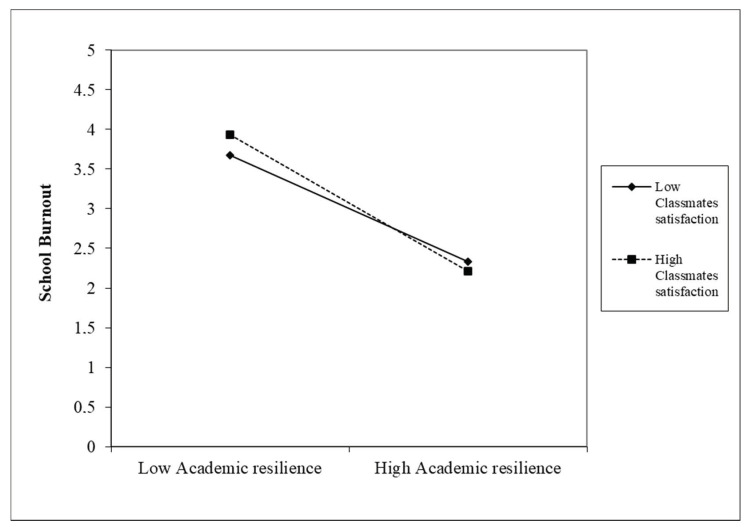
Moderation of satisfaction on the relationship with classmates on the link between academic resilience and school burnout.

The model that included gender, age, academic resilience, satisfaction on relationships with classmates, satisfaction on relationships with teachers, and the two interaction terms (academic resilience x satisfaction on relationships with classmates and academic resilience x satisfaction on relationships with teachers) explained 34% of the variance in school burnout [F(7, 568) = 42.887, *p* < 0.001]. The analysis of the β coefficients showed that age was significantly and positively associated with school burnout (β = 0.22, *p* < 0.001). Furthermore, academic resilience (β = −0.35, *p* < 0.001) and satisfaction on relationships with teachers (β = −0.30, *p* < 0.001) were significantly and inversely related to school burnout. Finally, satisfaction on relationships with classmates significantly moderated the effect of academic resilience on school burnout (β = −0.09, *p* < 0.05). In detail, the simple slope analysis (see Figure 1) highlighted that the inverse relationship between academic resilience and school burnout was more robust at higher levels (+1 SD; Simple slope = −1.06, SE = 0.09; 95% CI [−1.25, −0.86], *p* < 0.001) than at lower levels (−1 SD; Simple slope = −0.076, SE = 0.09; 95% CI [−0.95, −0.57], *p* < 0.001) of satisfaction on the relationships with classmates. The satisfaction on the relationships with teachers did not moderate the relationship between academic resilience and burnout.

## 4. Discussion

The current study sought to investigate the association among academic resilience, students’ satisfaction on the relationships with teachers and classmates, and school burnout in a sample of Italian high school students. Moreover, as the central core of the present study, we further examine the moderating role of satisfaction on the relationship with teachers and classmates in the link between academic resilience and school burnout. Findings partially supported the correlational and the moderation hypotheses.

In detail, results supported the association between academic resilience and school burnout by showing that, in our sample, they are inversely related. Our findings are in line with previous studies in the Italian context, showing that both in high school and university, students with higher academic resilience were able to counteract burnout better than their counterparts [25,26]. This datum could also be interpreted in light of Martin and Marsh’s definition of academic resilience [29]. According to the authors indeed, academic resilience is the capacity to overcome chronic and overwhelming school adversities and maladjustment, as school burnout is. Furthermore, as shown by previous studies (e.g., [33,38]), academically resilient students are more motivated and passionate about their studies than their lower resilient peers. Thus, it is possible to assume that these students, thanks to their resilient characteristics and the determination they employ to overcome obstacles, are better able to withstand the taxing pressures of the academic environment, being, therefore, more shielded against burnout.

Moreover, our findings highlighted that the more students are satisfied on the relations established with teachers, the less they feel burnt out. These results, echoing previous studies [41,43], underlined that students who perceive their learning environment as fulfilling and supportive are less inclined to experience their school path as unfavorable and hostile. Students who have a good relationship with their teachers are also more likely to communicate their difficulties and concerns about school (e.g., [39,42,43]). Thus, it is possible that knowing that they can count on a teacher who is understanding and attentive to their needs represents a source of additional security to effectively deal with the stressful and emotional burden related to the school demands.

In addition, our results showed no relation between the satisfaction on the relationship with classmates and school burnout. This unexpected finding could be interpreted in light of the role played by the peer group during adolescence. During this developmental stage, the peer group plays a fundamental role in the adolescent’s identity growth process. Its influence is often even more evident in extracurricular contexts, where norms shared are different from those imposed by the school system [62]. Therefore, it is possible that being satisfied on the relationships established with classmates does not in itself act as a protective factor against burnout, which, by its nature, is related to the demands of the school context. Accordingly, our results suggest that in the face of a chronic form of school maladjustment, what emerges as effectively protective is establishing a healthy relationship with teachers, who may potentially represent the origin of their discomfort. Another possible explanation could be found considering the quality of our studied variable. Classmates do not always correspond to the group in which an adolescent recognizes him/herself, who can identify with another peer group because of affinities and different communication codes. As shown by previous studies, indeed, identification in a peer group (other than classmates) with low academic achievement can strongly condition students’ burnout levels in the long run [21].

Furthermore, our findings did not support Hypothesis 2, revealing that the students’ satisfaction on the relationship with teachers did not moderate the relationship between academic resilience and burnout. In other words, the protective role of students’ academic resilience toward burnout is not affected by the different grades of their satisfaction in the relations established with teachers. This datum could be better understood in light of the nature of the teacher–student relationship. Unlike the relationship with classmates (i.e., horizontal), the teacher–student relationship is usually vertical. Thus, although the teacher may be a valuable source of support for the struggling student, he/she is generally at a different and higher level than pupils [39,63]. This difference, however, can also affect the way a student in his/her resilient process approaches the teacher to deal with his/her emotional difficulties related to the school context. Therefore, in counteracting burnout, academically resilient students may prefer or are conditioned by different sources of support, others than teachers (e.g., classmates).

In addition, our results show that satisfaction on the relationship with teachers is itself a protective factor toward burnout. Simply put, when students are remarkably fulfilled with their relationship with teachers, they do not need to rely further on their resilient characteristics to protect themselves from burnout. Effectively, they may feel already inserted in a highly satisfying and positive learning context [45].

Finally, our results supported Hypothesis 3, showing the moderating role of satisfaction on the relationship with classmates in the path of academic resilience and school burnout. Specifically, our results suggest that the protective role of academic resilience toward burnout is more robust at higher grades of satisfaction on the relationships established with classmates. In contrast, when the satisfaction on these relationships is low, the protective role of academic resilience on burnout is weakened. This datum, echoing previous findings (e.g., [54,55]), highlighted the importance of the classmates’ connectedness when dealing with challenging situations in the school context. When students are placed within a cooperative classroom climate with their classmates, they also feel freer to share daily difficulties and challenges. This experience of a highly interconnected and shared classroom environment increases the sense of belonging, facilitates motivational processes toward learning, and allows for a free expression of its emotional difficulties [52]. Despite this, as seen above from our data, being satisfied on the relationships with classmates does not act as a protective factor *per se* toward burnout. Still, it does serve as an enhancer of a resilient approach toward academic challenges. This finding aligns with previous studies demonstrating that high connection (and relationship satisfaction) with one’s classmates can mitigate perceptions of adverse events, fostering resilient processes [48,52]. In overcoming chronic difficulties related to the school environment, knowing that one can count on a cohesive class group becomes a source of support and sharing of the correct coping strategies to deal with traumatic school-related events [52,53,54].

### Limitations and Future Directions

The current study yields several limitations. Among them, the study’s cross-sectional nature did not allow us to draw causal relationships among the studied variables. Future longitudinal studies could help to better clarify the heart of the observed paths. Furthermore, despite our sample belonging to different Italy’s regions and various high schools, we did not consider these aspects in our analyses. In further studies, it would be interesting to compare the obtained results by splitting them based on the region of provenance and the high school attended.

Finally, it would be interesting to deepen the students’ satisfaction role, further specifying the different facets of the relationships with classmates and teachers.

## 5. Conclusions

The current study, consistent with the existing literature, highlights the role played by individual characteristics such as resilience in protecting students from extreme forms of maladjustment. Furthermore, it shows the importance of the school context and the relationships established within it. On the one hand, the satisfaction on the relationship with teachers acts itself as a highly protective factor; conversely, being satisfied on the relationship with classmates also affects the protective power of resilience towards burnout, interacting with it.

These results provide exciting suggestions for teachers, educators, and policymakers regarding practical interventions applied in classrooms to prevent maladjustment. First, our data provide important insights regarding the enhancement of the teacher–student relationship. A student who is satisfied on their relationship with their teachers is a student who is more confident in his/her abilities and better able to cope with academic adversity. Therefore, from the beginning of the academic year, teachers should pay attention not only to the purely notional aspect of teaching but should make a greater effort to connect with the students, giving weight and attention to their difficulties, fostering a classroom climate more devoted to sharing the problematic aspects of learning. Moreover, it could be efficient to work on burnout prevention by focusing on the synergetic empowerment of both resilience and classroom relationships and giving specific regard to student–student interactions. In practical terms, and based on our results, principals and teachers could add, to the regular class hours, periodic extra-curricular activities with the class group to cement more the relationship between classmates. In addition, and in line with previous statements, the school board could officially establish moments of exchange between classmates at the end of lessons, coordinated and monitored by teachers, to share and exchange both the difficulties encountered in studying and the most effective coping strategies that have been adopted to deal with them. Creating a synergy between the class group and teachers opens up the possibility of structuring a class that acts as a resilient community where motivational and emotional difficulties are periodically addressed, and the resilient characteristics of individuals are enhanced.

## Figures and Tables

**Table 1:** Descriptive statistics and correlation matrix.

Variables	M	SD	Min	Max	Skewness	Kurtosis	2	3	4	5	6
1. Gender	-	-	-	-	-	-	-	0.004	−0.105 *	0.142 **	0.019
2. Age	15.73	1.56	14	18	0.217	−1.49		−0.127 **	−0.224 **	−0.157 **	0.225 **
3. AR	23.40	4.70	7	35	−0.278	−0.023			0.284 **	0.410 **	−0.485 **
4. SC	3.10	0.79	1	4	−0.642	0.049				0.240 **	−0.185 **
5. ST	2.79	0.66	1	4	−0.559	0.673					−0.457 **
6. SBI	27.93	9.05	9	52	0.212	−0.424					

Note. AR = Academic resilience; SC = Students’ satisfaction on the relationship with peers; ST = Students’ satisfaction on the relationship with teachers; SBI = School burnout; * *p* < 0.05; ** *p* < 0.01.

**Table 2:** Multiple hierarchical regression: moderations of perceived students’ satisfaction on relationships with classmates and teachers on the relationship between academic resilience and school burnout.

Outcome: School Burnout	β	ΔR^2^	95% CI
Step 1		0.051 **	
Gender	0.014		−1.195, 1.705
Age	0.22 **		0.841, 1.770
Step 2		0.028 **	
AR	−0.35 **		−0.817, −0.528
ST	−0.30 **		−5.220, −3.160
SC	0.025		−0.540, 1.121
Step 3		0.008 *	
AR × ST	−0.010		−0.221, 0.167
AR × SC	−0.090 *		−0.356, −0.038

Note. * *p* < 0.05; ** *p* < 0.01; ΔR^2^ = R-squared changes; CI = Confidence interval; AR = Academic resilience, ST = Students’ satisfaction on the relationship with teachers, SC = Students’ satisfaction on the relationship with peers, AR × ST = Interaction term between AR and ST, AR × SC = Interaction term between AR and SC.
